# Nociceptors regulate osteoimmune transcriptomic response to infection

**DOI:** 10.1038/s41598-023-44648-9

**Published:** 2023-10-16

**Authors:** Katherine V. Lillis, Obadah Austah, Ruta Grinceviciute, Gustavo P. Garlet, Anibal Diogenes

**Affiliations:** 1 Department of Endodontics, University of Texas Health at San Antonio, San Antonio, TX 78229 USA; 2 Present Address: Department of Endodontics, Faculty of Dentistry, King Abdulaziz University, Jeddah, Saudi Arabia; 3 Department of Biological Sciences, Bauru School of Dentistry, University of São Paulo, São Paulo, Brazil

**Keywords:** Neuroimmunology, Osteoimmunology

## Abstract

Osteoimmune diseases, such as apical periodontitis, are prevalent, often painful, inflammatory conditions resulting in bone loss and reduced quality of life. There is growing evidence that the nociceptive fibers densely innervating affected tissues regulate disease progression; therefore, we hypothesized that nociceptors regulate the transcriptomic profile of the periapical osteolytic lesion in a mouse model of apical periodontitis. Male control and nociceptor-ablated mice underwent pulp exposures, and after 0, 7, or 14 days, total RNA from periapical tissues was submitted for sequencing and bioinformatic analysis. Pulp exposure triggers the differential expression of hundreds of genes over the course of infection. At 14 days post pulp exposure, 422 genes, including Tnf, Il1a, and Il1b, were differentially expressed between nociceptor-ablated and control mice with greater enrichment of biological processes related to inflammation in nociceptor-ablated mice. Nociceptor ablation regulates the transcriptomic profile of periapical lesions in a mouse model of apical periodontitis, shifting the gene expression profile to a greater enrichment of inflammatory genes, suggesting nociceptors play a role in the kinetics of the immune response. This newly uncovered neuro-immune axis and its mechanisms in apical periodontitis can be an important therapeutic target for the treatment of this prevalent disease.

## Introduction

Osteoimmune diseases, such as apical periodontitis, are prevalent, often painful, inflammatory conditions resulting in bone loss and reduced quality of life^1^. There has been extensive work examining the osteo-immune axis to further understand interactions between immune cell activity and bone metabolism^2–5^. In addition to immune and bone metabolism cells, inflammatory sites in these diseases are richly innervated by nociceptors, a subclass of sensory neurons commonly known to drive pain signaling in the body^6–9^. Notably, there is growing evidence nociceptors are active in regulating immune responses, as they are activated by inflammatory mediators and noxious stimuli from invading bacteria^3,6–12^. In such an environment, immune cells are known to secrete inflammatory mediators such as cytokines, chemokines, prostaglandins, and proteolytic enzymes, leading to osteolysis^5^. Although a direct relationship between the increase in inflammatory mediators and osteolysis has been demonstrated, far less is known about how nociceptors regulate these inflammatory processes in the context of bone loss due to root canal infections.

As such, we have recently shown that in a rodent model of apical periodontitis (AP), nociceptors are osteoprotective by attenuating bone loss after infection when compared to nociceptor-ablated rodents^8,9^. We found that nociceptor ablation results in an earlier increase in inflammation, as shown by the upregulation of key cytokines and a greater influx of macrophages and lymphocytes. In addition, we have shown that nociceptors directly regulate osteoblastic and osteoclastic functions, demonstrating direct communication with bone metabolism cells^8^. While the progression of AP inflammatory responses had been previously characterized^13^, the overall transcriptomic profiling of this process and its regulation by nociceptors is unknown. Therefore, in this study, our objective was to employ the transgenic ablation of nociceptors and transcriptomic analysis to determine biological pathways regulated by nociceptors in apical periodontitis.

## Results

### Pulp exposure results in the differential expression of genes within the periapical lesion over 14 days in both nociceptor-ablated and control mice

**Figure 1:**
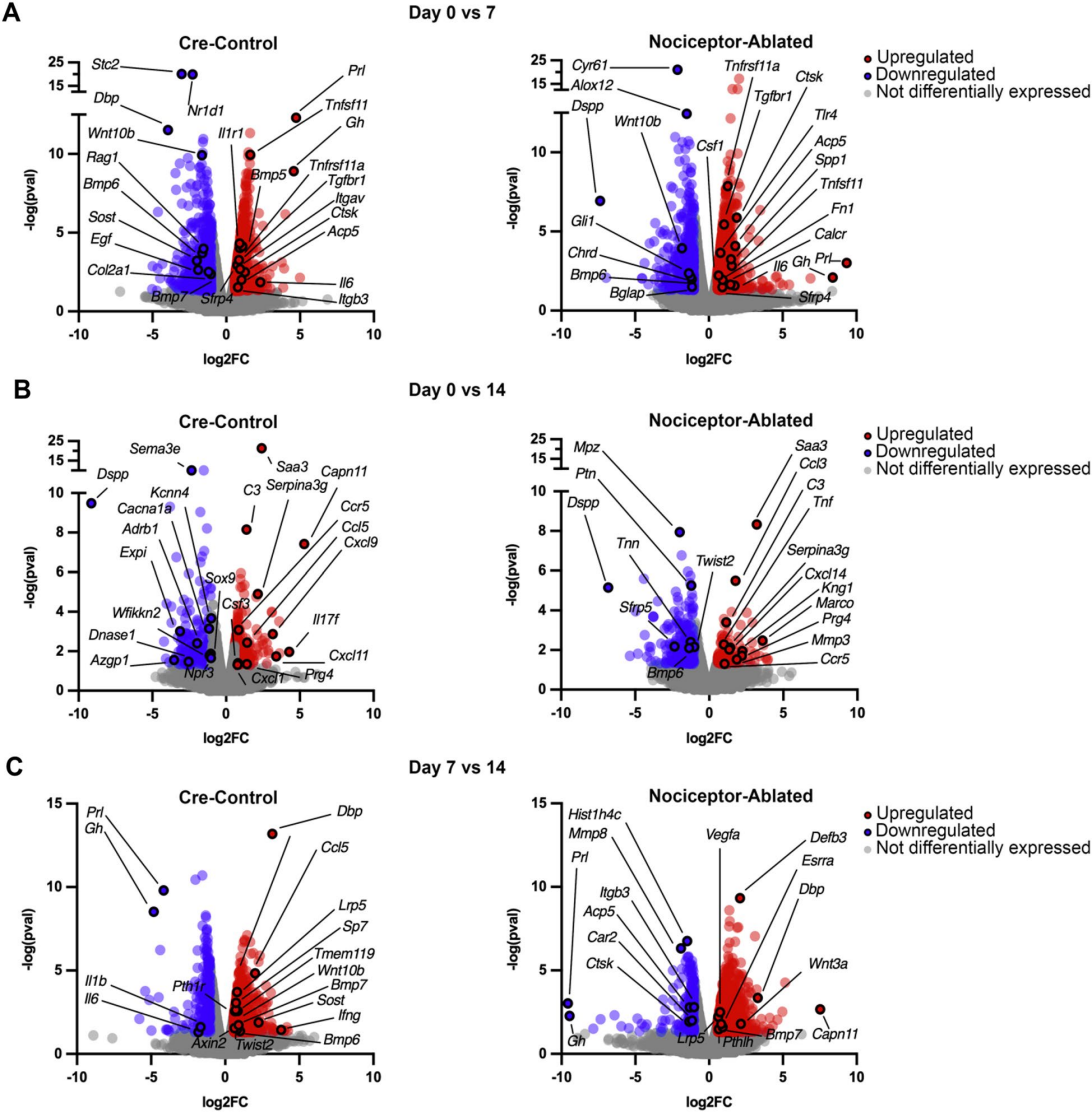
Pulp exposure results in the differential expression of genes within the periapical lesion over the course of 14 days in nociceptor-ablated and control mice. Volcano plots of differentially expressed genes (DEGs) in periapical lesions compared between 0- and 7-days (**A**), 0- and 14-days (**B**), and 7- and 14-days (**C**) following pulp exposure in cre-control and nociceptor-ablated mice, where log_2_(fold change [FC]) is plotted against –log(pval) (n = 3–4 mice/strain/time point). The R package ‘DESeq’ was used to normalize data and find group-pairwise differential gene expression.

After 7 days of infection, both nociceptor-ablated and cre-control mice exhibited hundreds of differentially expressed upregulated and downregulated genes (fold change [FC] > 1.5 and *p* < 0.05) compared to Day 0 (Fig. 1A). Within upregulated genes, nociceptor-ablated mice had a greater FC for genes, including osteolytic-specific genes Acp5, Ctsk, and Tnfrsf11a. Nociceptor-ablation also resulted in a greater downregulation of Wnt10b compared to control. However, control mice had a greater initial upregulation of Tnfsf11 and IL6 as well as a greater downregulation of Bmp6. After 14 days of infection, nociceptor-ablated mice had a greater fold change increases in upregulated genes including Prg4, Saa3, and Ccr5 compared to control (Fig. 1B). Conversely, control mice exhibited a greater upregulation of C3 and Serpina3g and a greater downregulation of Dspp. Between 7 and 14 days of infection, nociceptor-ablated mice showed greater upregulation of Dbp and Bmp7, whereas control mice showed greater upregulation of Lrp5 (Fig. 1C). Interestingly, nociceptor-ablated mice showed a greater downregulation of Cthrc1, while control mice had greater downregulation of Ccr1.

### Nociceptor-ablated mice exhibit unique up- and downregulated genes compared to control in addition to significantly greater expression of inflammatory genes at each timepoint

**Figure 2:**
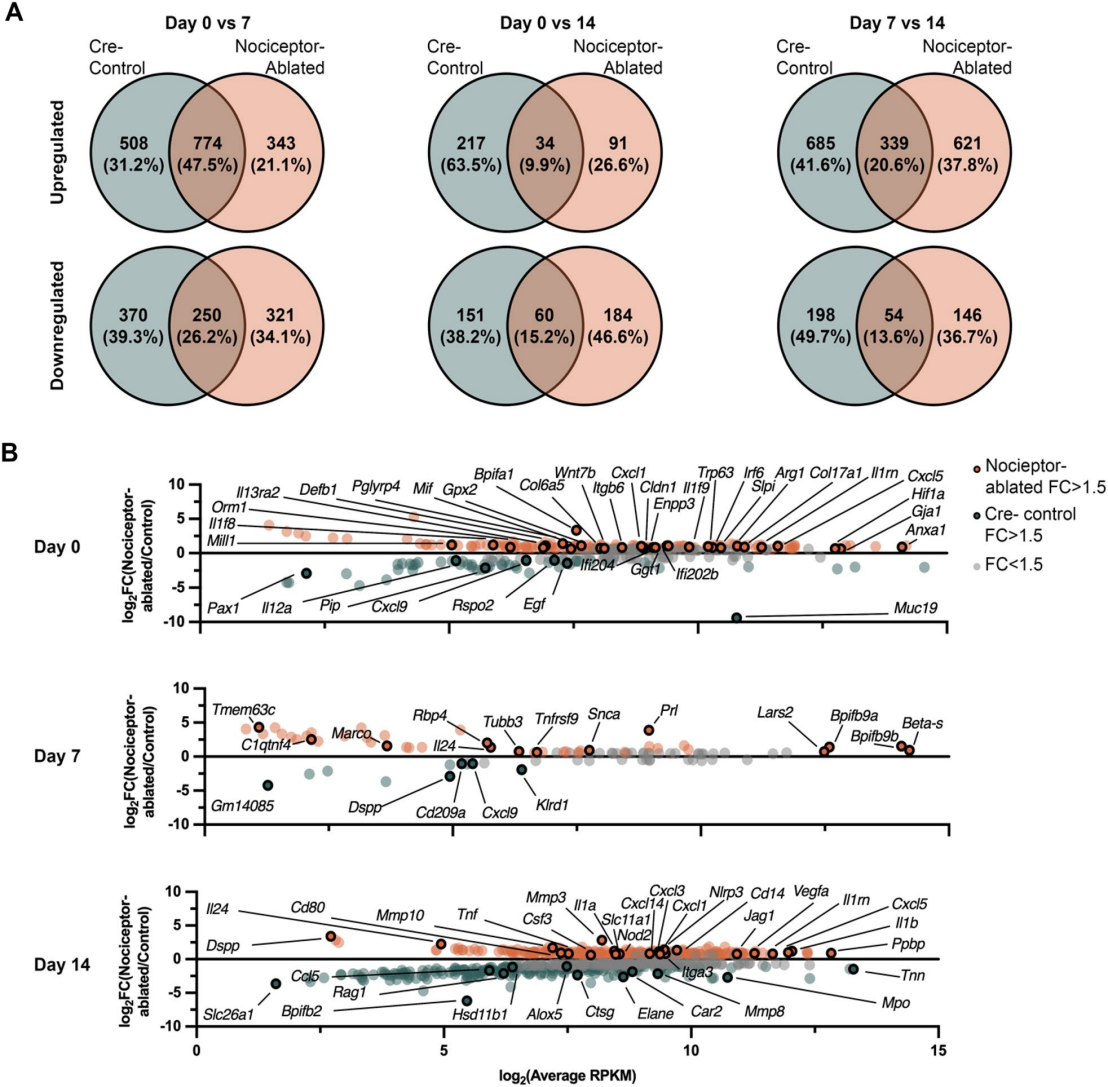
Nociceptor-ablated mice exhibit unique differentially expressed genes throughout the progression of apical periodontitis compared to cre-control. Venn diagrams of number of up- or down-regulated DEGs and percent of total up- or down-regulated DEGs for nociceptor-ablated and cre-control mice between 0 and 7, 0 and 14, and 7 and 14 (**A**). DEGs between cre-control and nociceptor-ablated at Days 0, 7, and 14 are shown (**B**). DEGs were defined as genes where *p* < 0.05, and FC > 1.5 (n = 3–4 mice/strain/time point). The R package ‘DESeq’ was used to normalize data and find group-pairwise differential gene expression.

Over the course of the infection, nociceptor-ablation resulted in unique changes in gene expression. Within the first 7 days, 47.5% of upregulated genes and 26.2% of downregulated genes were shared between nociceptor-ablated and control mice (Fig. 2A). From Day 0 to Day 14, 9.9% of upregulated and 15.2% of downregulated genes were shared among the strains Fig. 2A). Moreover, from Day 7 to Day 14, 20.6% of upregulated and 13.6% of downregulated genes were common between nociceptor-ablated and control mice (Fig. 2A).

Of all the differentially expressed genes at each experimental time following infection, the ratio of relative enrichment between nociceptor-ablated and control mice was determined. At Day 0, 155 genes had significantly higher expression in nociceptor-ablated mice (e.g., Gja1, Hif1a, Cxcl1, Cxcl5, and Mif) compared to control (FC > 1.5; *p* < 0.05), and 57 genes that had significantly lower expression in control mice (e.g., Rspo2, Cxcl9, and Pax1) (FC > 1.5 and *p* < 0.5 and) (Fig. 2B). At 7 days post-pulp exposure, there were 43 genes with significantly greater expression in nociceptor-ablated mice (e.g., Marco, Snca, and Tnfrsf9) and 10 genes with significantly greater expression in control mice (e.g., Cxcl9, Dspp, and Klrd1) (Fig. 2B). After 14 days of infection, there were 257 genes with greater expression in nociceptor-ablated mice (e.g., Vegfa, IL1a, IL1b, and Tnf) compared to 219 genes with greater expression in control mice (e.g., Alox5, Car2, Ccl5, Elane, and Mmp8) (Fig. 2B).

### Nociceptor-ablation results in a greater enrichment of biological processes related to inflammation, specifically immune cell chemotaxis and migration, compared to cre-control after 14 days of infection

**Figure 3:**
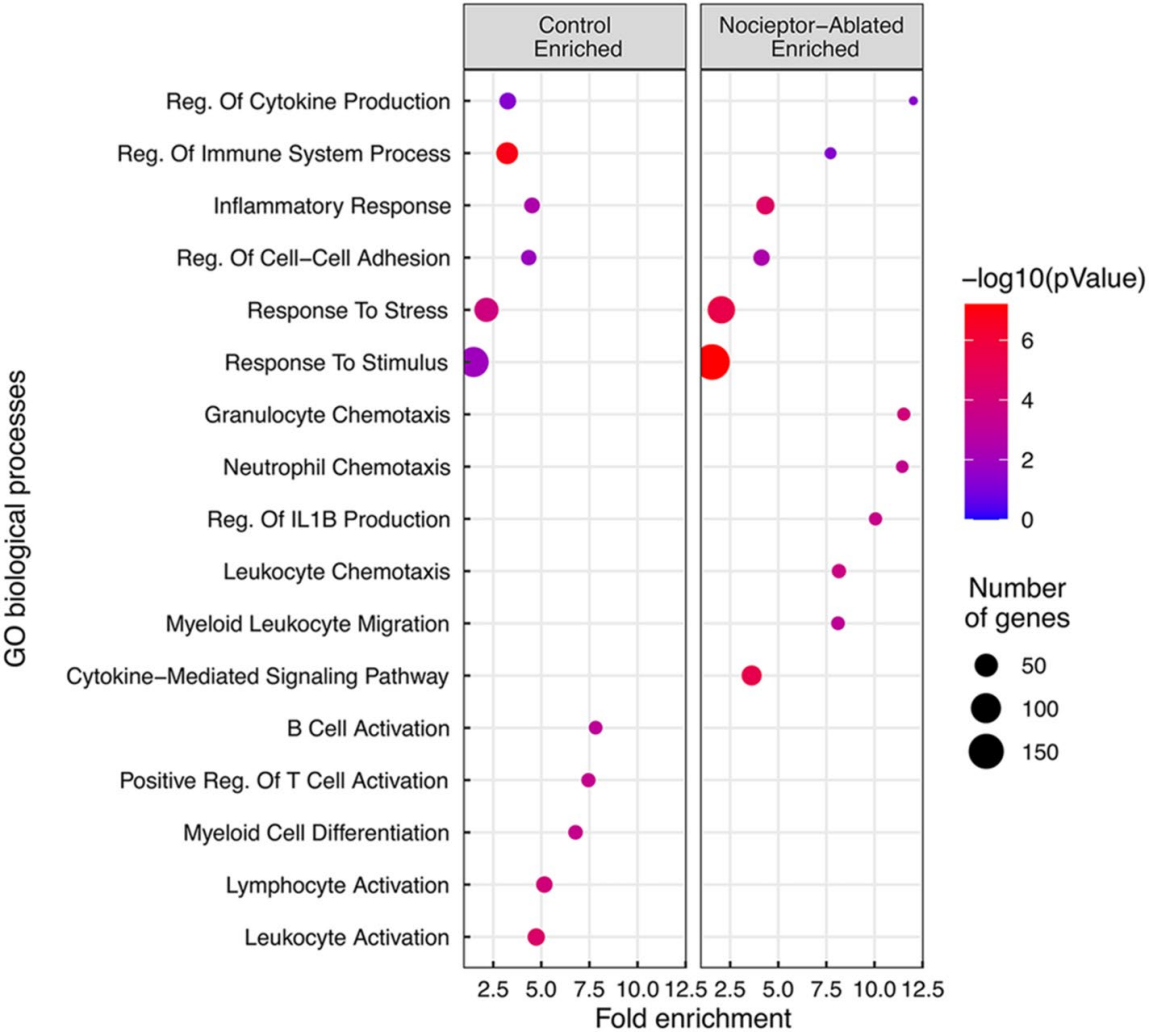
Nociceptor-ablation results in a greater enrichment of biological processes related to inflammation, specifically immune cell chemotaxis and migration, compared to cre-control after 14 days of infection. Enriched gene ontology (GO) biological processes of DEGs between cre-control and nociceptor-ablated lesions at 14 days of infection are plotted as Fold Enrichment for each biological process, where color correlates to -log(*p*Value) and size corresponds to the number of genes enriched in each process.

After 14 days following pulp exposure, 257 genes were significantly greater expressed in nociceptor-ablated mice, whereas 219 genes were significantly reduced expressed in ablated mice. Gene ontology analysis revealed biological processes enriched within each set of differentially expressed genes (Fig. 3). Among the biological processes enriched in both nociceptor-ablated and control mice, “Regulation of Cytokine Production” was ~ 3.7 times more enriched in nociceptor-ablated mice. Similarly, “Regulation of Immune System Process” was ~ 2.4 times more enriched in these mice compared to control. Furthermore, the number of genes within overlapping biological processes was greater in nociceptor-ablated mice, including “Inflammatory Response”, “Regulation of Cell–Cell Adhesion”, “Response to Stress”, and “Response to Stimulus”.

Moreover, subsets of biological processes were unique to either nociceptor-ablated or control differentially expressed genes. Within nociceptor-ablated mice, biological processes including “Granulocyte Chemotaxis,” “Neutrophil Chemotaxis,” and “Regulation of IL-1β Production” were enriched. Biological processes that were only enriched in control mice include “B Cell Activation,” “Positive Regulation of T Cell Activation,” and “Myeloid Cell Differentiation.”

### After 14 days of infection, nociceptor-ablated lesions display unique patterns of expression of genes related to bone metabolism, immune cell, and inflammatory processes

**Figure 4:**
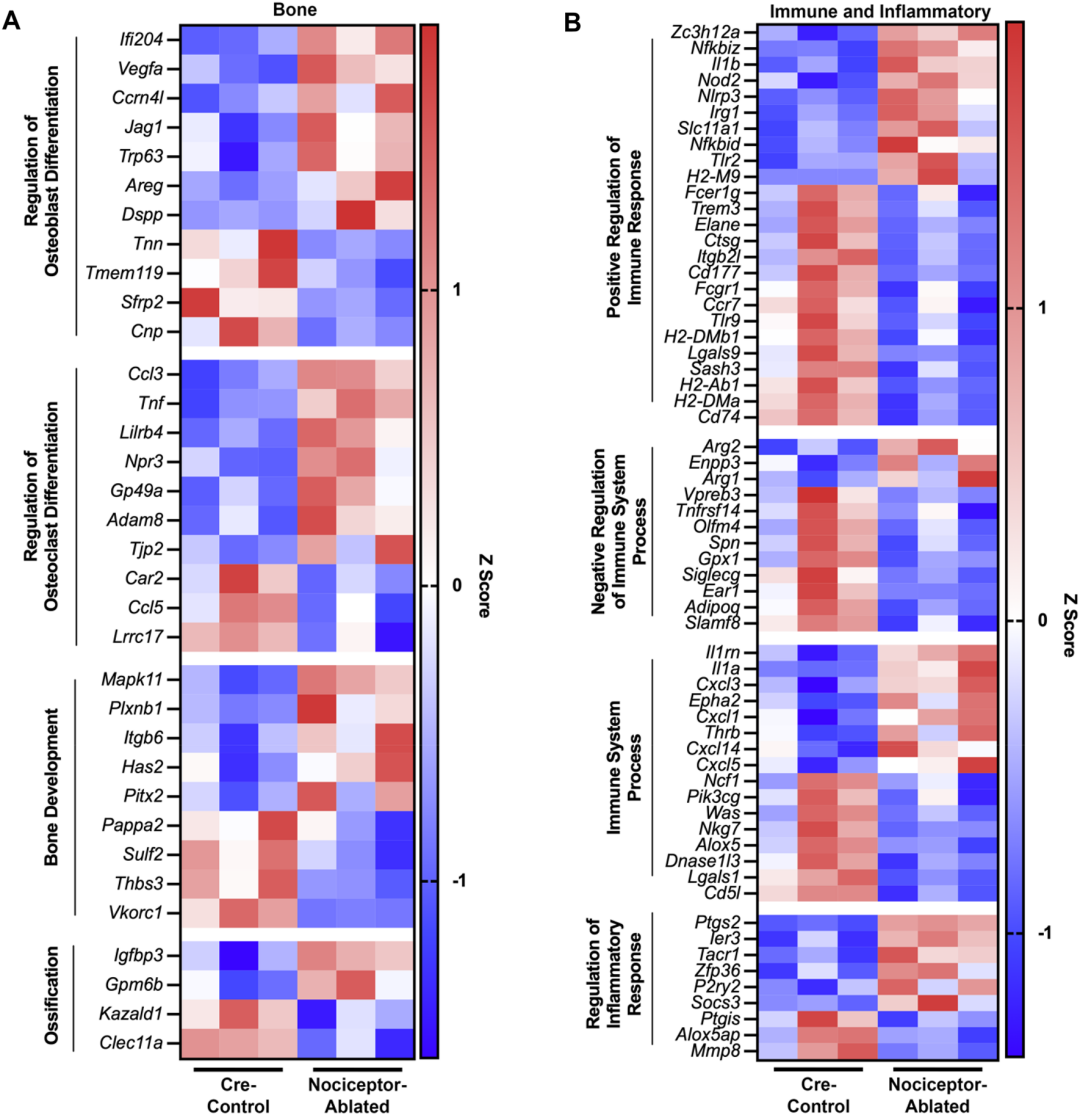
After 14 days of infection, nociceptor-ablated lesions display unique patterns of expression of genes related to bone, immune, and inflammatory processes. Heatmap of select inflammatory DEGs expressed in the periapical lesions from mice on Day 14 post- pulp exposure related to bone (**A**) and immune and inflammatory (**B**) biological processes. Color corresponds to z score of Reads Per Kilobase of transcript per Million mapped (RPKM) values of each gene (n = 3 mice/strain).

Within periapical lesions following 14 days of infection, nociceptor-ablation modulates expression of genes related to bone biology, particularly in processes related to regulation of osteoblast differentiation (e.g., Vegfa, Dspp, and Sfrp2), regulation of osteoclast differentiation (e.g., Ccl3, Tnf, and Ccl5), bone development, and ossification (Fig. 4A). Among genes related to immune and inflammatory processes, nociceptor-ablation modulates expression of genes within positive regulation of the immune response (e.g., Zc3h12a, Nod2, Nlrp3, IL1b, Nfkbid, and Slc11a1) and inflammatory response (e.g., Zfp36, Ptgs2, Alox5, Alox5ap, and Ptgis) (Fig. 4B).

### Nociceptor-ablated mice show greater expression of genes known to modulate development and progression of apical periodontitis throughout the course of infection

Throughout the course of the infection, nociceptors modulate the expression of various genes within the periapical lesion. At 14 days following pulp exposure, nociceptor-ablated mice exhibit greater levels of genes including Ccl3, IL1a, IL1b, Junb, Mmp3, and Tnf (Supplementary Fig. S1-3). Conversely, control mice exhibit greater expression of Ccl5, Mmp8, Sfrp2, Tmem119, and Tnn after 14 days of infection. Some genes are differentially expressed at multiple time points, including Dspp, Nod2, and Vegfa.

We also examined genes for known markers of immune cells and found nociceptor-ablated mice had significantly lower expression of B cell marker, Cd19, and M2 Macrophage marker, Cd163, after 14 days of infection compared to cre-control mice (Supplementary Fig. S4). Expression of genes within the periapical lesion after 14 days of infection was confirmed with real time PCR, where nociceptor-ablation resulted in a significant upregulation of IL1a (*p* = 0.01), Tnf (*p* = 0.01), and Nlrp3 (*p* < 0.01), whereas ablated mice had reduced expression of Alox5 (*p* = 0.03) (Supplementary Fig. S1). Ccl3 and Dspp expression were not significantly different between genotypes, (*p* = 0.07 and* p* = 0.22, respectively) but Dspp expression in nociceptor-ablated mice decreased ~ 515 fold compared to Day 0 and decreased ~ 320 fold in cre-control mice.

### Discussion

The crosstalk between bone and neural tissue has been a growing area of research interest^14^, yet there has been a lack of studies investigating this communication in the context of an inflammatory environment. Despite an existing body of evidence on some of the molecular events that govern the initiation and maintenance of AP, this study is the first to establish the transcriptomic profiling underlying its pathogenesis. Moreover, we have demonstrated that nociceptive neuronal fibers innervating the tissues surrounding teeth, through multiple cell-to-cell interactions, modulate this disease progression^8^. In this study, we have found that nociceptors regulate the transcriptomic profile of periapical lesions in a mouse model of AP. Specifically, nociceptor-ablation enriches the expression of inflammatory genes and biological processes after 14 days of infection, suggesting nociceptor-ablation impacts the kinetics of the immune response in AP. Periapical lesions that develop in AP comprise a complex inflammatory cascade in response to infection, consequently contributing to osteolytic activity and bone loss. Therefore, investigating mechanisms targeting these inflammatory processes has tremendous potential for developing novel AP treatments.

#### Effects of nociceptors on bone metabolism genes

Among the hundreds of genes differentially regulated throughout the course of the infection in AP, many were involved in bone metabolism. Notably, nociceptor-ablation resulted in a greater upregulation of genes that promoted osteolytic activity by either promoting osteoclastogenesis (i.e., Acp5, Ctsk, Tnfrsf11a, Ccr5) or inhibiting osteoblastogenesis (i.e., Saa3)^15–17^. Additionally, key mineralization genes that were initially downregulated in control mice from Days 0 to 7 (i.e., Wnt10b, Bmp6, and Sost) were upregulated between Day 7 and 14, suggesting a potential mineralization repair attempt seen in control but not nociceptor-ablated mice, matching patterns of AP bone loss we have observed at 14 days^8^. Differences in the expression of key genes between strains were confirmed with RT-PCR, further validating the enriched osteolytic profile in nociceptor-ablated mice. The dramatic decrease in Dspp expression shown in both RNA sequencing and RT-PCR illustrates the osteolytic nature of the disease, with a strong inhibition of mineralization. Moreover, Junb, a component of the transcription factor complex activated by RANK/RANKL binding, is expressed greater in nociceptor-ablated mice, suggesting greater osteoclast differentiation^15^. Within the process of tissue degradation, matrix metalloproteinases (MMPs) play a key role. As such, Mmp3 has greater expression in nociceptor-ablated mice, whereas Mmp8 is expressed greater in control mice, suggesting nociceptors differentially regulate expression of these enzymes throughout the course of infection^18,19^. Other genes involved in promoting osteolytic activity include Nod2 and Vegfa, which had greater expression in nociceptor-ablated mice^20,21^ Overall, these findings suggest nociceptors play a strong role in protecting against AP bone loss by decreasing the expression of genes promoting osteoclastogenesis and promoting the expression of those related to mineralization.

#### Effects of nociceptors on inflammatory pathways

Nociceptor-ablation resulted in greater enrichment of genes related to cytokine production and immune system processes compared to control after 14 days of infection. Moreover, differentially expressed genes in nociceptor-ablated mice were enriched in processes involved in immune cell chemotaxis and migration, whereas control differentially expressed genes were enriched in immune cell differentiation and activation. We have previously shown that nociceptor-ablation results in greater infiltration of immune cells earlier in the infection^8^, which is recapitulated in the current study examining immune cell marker expression. These results suggest nociceptors play an inhibitory role in the kinetics of immune cell differentiation, activation, and migration, which could contribute to their suppression of excessive AP bone loss. Indeed, nociceptor-ablated mice had significantly greater expression of inflammatory and bone metabolism genes compared to control mice. Particularly, IL1a, IL1b, and Tnf expression were greater in the lesions from nociceptor-ablated mice. These mediators are known to regulate the inflammatory response and aid in the differentiation of osteoclasts^5^. In addition, many of the genes upregulated in nociceptor-ablated mice are associated with inflammasome pathway signaling, including Nod2, Nlrp3, and IL1b^22^. Inflammasome signaling subsequently activates nuclear factor-κB signaling and transcription of inflammatory mediators, particularly cytokines^22^. The increased expression of Zc3h12a and Nfkbid in nociceptor-ablated mice further supports this mechanism, as these genes are key elements of the nuclear factor-κB pathway^23,24^. It is noteworthy that a shift to an anti-inflammatory M2 macrophage (CD163) phenotype seen in control mice was inhibited in the nociceptor-ablated mice. Future studies can further investigate direct mechanisms through which nociceptors modulate inflammasome pathways within AP lesions.

#### Limitations of study

While these results provide insight into biological processes regulated by nociceptors in apical periodontitis within the inflammatory site, there are a few limitations to this approach. Although the sample size of three to four biological replicates per genotype and time point is consistent with other transcriptomic studies^25^, greater sample sizes could result in an even more robust transcriptomic analysis. Although high-throughput sequencing is a powerful bioinformatic tool, we ensured the reproducibility of our results by repeating these experiments in additional mice, validating changes in genes of interest using real-time PCR. It is important to highlight that transcriptomic changes may not represent changes in protein expression or functional changes. Nonetheless, the results presented in this study are the first insight on transcriptomic changes in the kinetics of apical periodontitis development and how nociceptors regulate it.

#### Conclusion

Taken together, these results further emphasize the complex nature of changes that occur during the initiation, development of maintenance of apical periodontitis, and how nociceptors regulate the disease. They directly affect the activity of bone metabolism cells and modulate immune response profile, at least in part, through a global change in transcriptomics. Although AP is marked by pain leading to loss of function and lowered quality of life, the results of this study further confirm that the dense innervation present in tissues surrounding teeth does not solely serve a sensorial function. This newly uncovered neuro-immune axis and its mechanisms in AP can be an important therapeutic target for the treatment of this prevalent disease.

## Materials and methods

### Animals

Mice were bred and housed in a light/temperature-controlled environment at UT Health San Antonio with 2–5 animals per cage where food and water were available ad libitum. Male mice aged 8–12 weeks were used for all experiments, and nociceptor-ablated mice were generated as described previously^26^. Briefly, heterozygous Nav1.8^cre+^ mice were crossed with homozygous Rosa26^tm1 (DTA)Lky+^ to produce cre-control (Nav1.8 ^cre+/−^) and nociceptor-ablated (Nav1.8 ^cre+/−^ DTA^lox+/−^) mice. Mice were genotyped and underwent capsaicin eye-wipe nocifensive behavioral testing to confirm ablation and include in study, as described previously^8^. All studies were approved by the Institutional Animal Care and Use Committee of the University of Texas Health Science Center at San Antonio (Protocol 20170053AR). Furthermore, all experiments were performed in accordance with the National Institutes of Health Guide and Public Health Service policy on humane care and use of laboratory animals and ARRIVE guidelines of the National Center for the Replacement and Reduction of animals in Research.

### Apical periodontitis model and RNA isolation

Apical periodontitis was induced by performing pulp exposure procedures and allowing infections to occur for either 0 (n = 3 mice/strain), 7 (n = 4 mice/strain), or 14 days (n = 3 mice/strain) (n = 20 mice total; samples sizes derived from^8^). Briefly, an intraperitoneal injection of ketamine (75 mg/kg)/dexdomitor (1 mg/kg) was given to anesthetize mice, as described previously^9^. The mice were then mounted on a custom jaw retraction apparatus, and pulp exposures were created in all four first molars, and an intraperitoneal injection of Antisedan (1 mg/kg) was given to reverse the anesthesia. Some mice were immediately euthanized via cervical dislocation following pulp exposure (Day 0 group), while the remaining were transferred were returned to the housing facility. At the appropriate time point (Day 7 or Day 14 post-pulp exposure), mice were briefly anesthetized with isoflurane and euthanized via cervical dislocation. Then the first molars were dissected, pooling 4 teeth per mouse, and immediately snap frozen in liquid nitrogen and stored at -80ºC. After tissue grinding with a frozen surgical blade and homogenization in RNA lysis buffer, total RNA was isolated from harvested periapical lesions using the RNAeasy Kit (Qiagen) according to the manufacturer’s recommendation. After quantification with Nanodrop (Thermo Scientific), RNA was stored at − 80 °C.

### RNA sequencing

Total RNA was then submitted to the Genome Sequencing Facility at UT Health San Antonio for RNA sequencing and blinded bioinformatic analysis^25^. Total RNA was used for RNA-Seq library preparation by following the KAPA Stranded RNA-Seq Kit with RiboErase (HMR) sample preparation guide (Cat. #, KR1151, KAPA Biosystems). All RNA sequencing FastQ reads were aligned with the reference genome (UCSC mouse build mm9) using TopHat2 default settings (v2.0.8)^27^. The aligned BAM files were sorted (SAMTools) and then processed using HTSeq-count (v.0.6.0)^28^ to obtain the counts per gene, and Reads Per Kilobase of transcript, per Million mapped reads (RPKM) were calculated.

### Real time PCR

For validation with real time PCR at either day 0 or 14 (n = 7 mice/strain/day, n = 28 mice total), cDNA was first synthesized from total RNA using the High-Capacity RNA-to-cDNA kit according to the manufacturer’s instructions (Thermo Fisher Scientific). Then, the Taqman™ Fast Advanced Master Mix (Thermo Fisher Scientific) and Taqman™ gene expression assays were used to conduct real time PCR experiments on an ABI 7500 Fast Real-Time PCR System (Thermo Fisher Scientific). The comparative delta-delta cycle threshold method (ΔΔCt) was used to determine the relative expression for each gene of interest normalized to GAPDH, using Day 0 samples as the calibrator, as previously described^29^. We used the following Taqman target probes: Gapdh (Mm99999915_g1), Il1a (Mm00439620_m1), Tnf (Mm00443258_m1), Ccl3 (Mm00441259_g1), Ccl5 (Mm01302427_m1), Nlrp3 (Mm00840904_m1), Alox5 (Mm01182747_m1), and Dspp (Mm00515667_g1).

### Experimental design and statistical analysis

For RNA sequencing analysis, the R package ‘DESeq’ was used to normalize data and find group-pairwise differential gene expression, and comparisons were performed within strains for Day 0 versus 7, Day 0 versus 14, and Day 7 versus 14^25^. Comparisons were also performed between strains at Day 0, Day 7, and Day 14. Differentially expressed genes (DEGs) were defined as Fold Change (FC) > 1.5 and *p* < 0.05. For Day 14 DEGs, gene ontology biological process analyses were performed using Protein Analysis Through Evolutionary Relationships (PANTHER) Overrepresentation Test (PANTHER 16.0 Released 20,210,224)^30^. Briefly, Fold Enrichment scores for enriched Gene Ontology Biological Processes enriched with DEGs compared to the *Mus musculus* reference list were generated along with number of genes present in the annotation data category and p-values using a Fisher test and Bonferroni correction^31^. Statistical analysis for real time PCR was performed using unpaired t tests. Statistical significance set at *p* < 0.05. All statistical analyses were performed using GraphPad Prism 7.0 (GraphPad). The datasets generated for this study can be found in the Gene Expression Omnibus (GEO) #GSE205195.

### Supplementary Information

Supplementary Information.

## Data Availability

The datasets generated for this study can be found in the Gene Expression Omnibus (GEO) #GSE205195 with the token “avydwcuyrlwnzgx”.
