# Supplementary material for: Nociceptors regulate osteoimmune transcriptomic response to infection
Source: Sci Rep. 2023 Oct 16;13:17601. doi: 10.1038/s41598-023-44648-9 (PMC10579402; doi:10.1038/s41598-023-44648-9)
Supplement: Supplementary file 1 — Supplementary Information. [file 41598_2023_44648_MOESM1_ESM.docx]

##
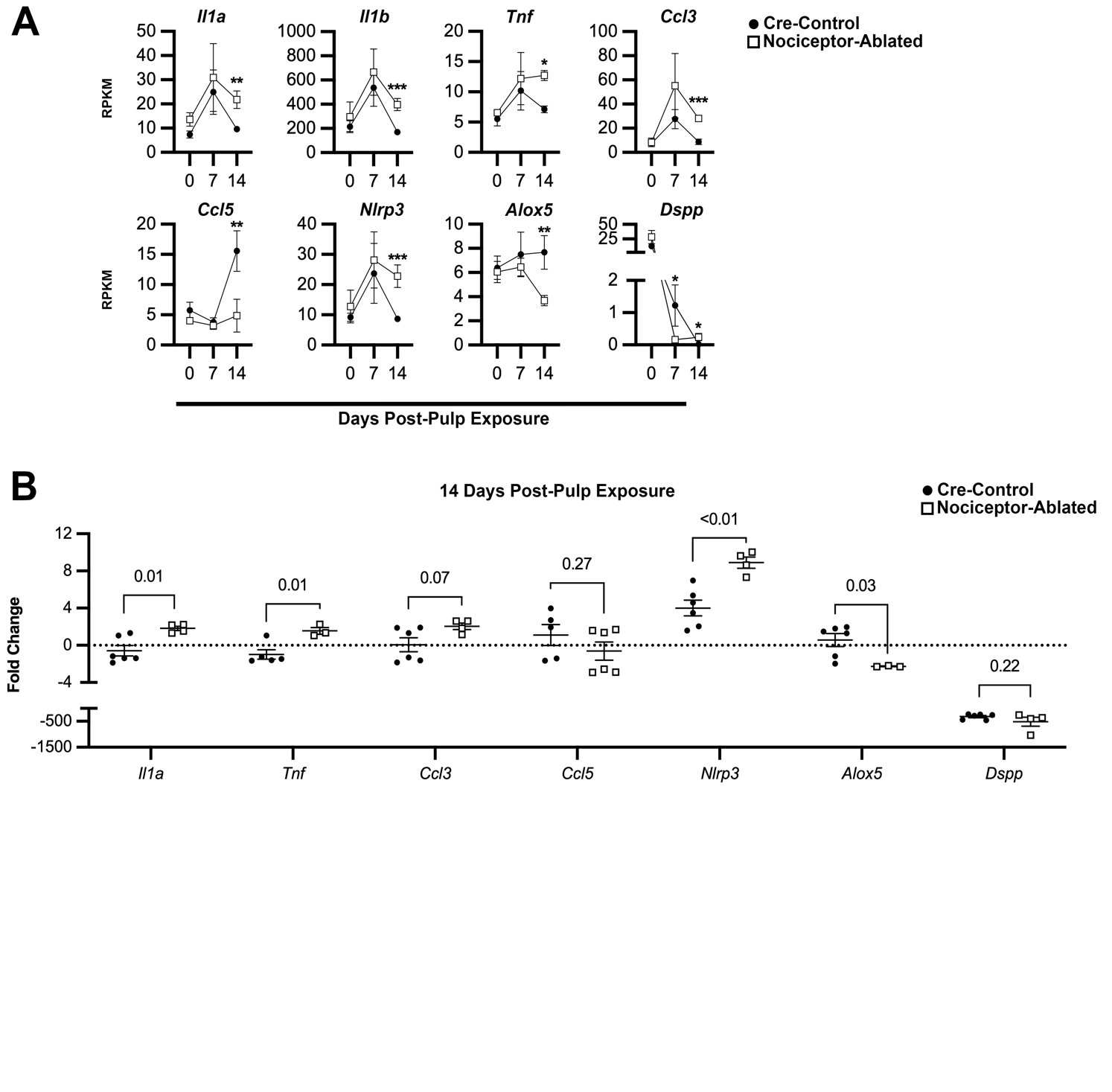


## Supplementary Figure S1 Nociceptor-ablated mice show greater expression of genes known to modulate development and progression of apical periodontitis throughout the course of infection.

Individual expression of selected genes at 0, 7, and 14 days of infection are presented as RPKM (n=3-4 mice/strain) (A). The R package ‘DESeq’ was used to normalize data and find group-pairwise differential gene expression. Validation of RNA sequencing results with real time PCR after 14 days of infection, where data are presented as fold change of respective Day 0 gene expression (n=3-7 mice/strain), and data represent mean ± SEM as analyzed with unpaired t-test (B). *p<0.05, **p<0.001, ***p<0.0001.

**
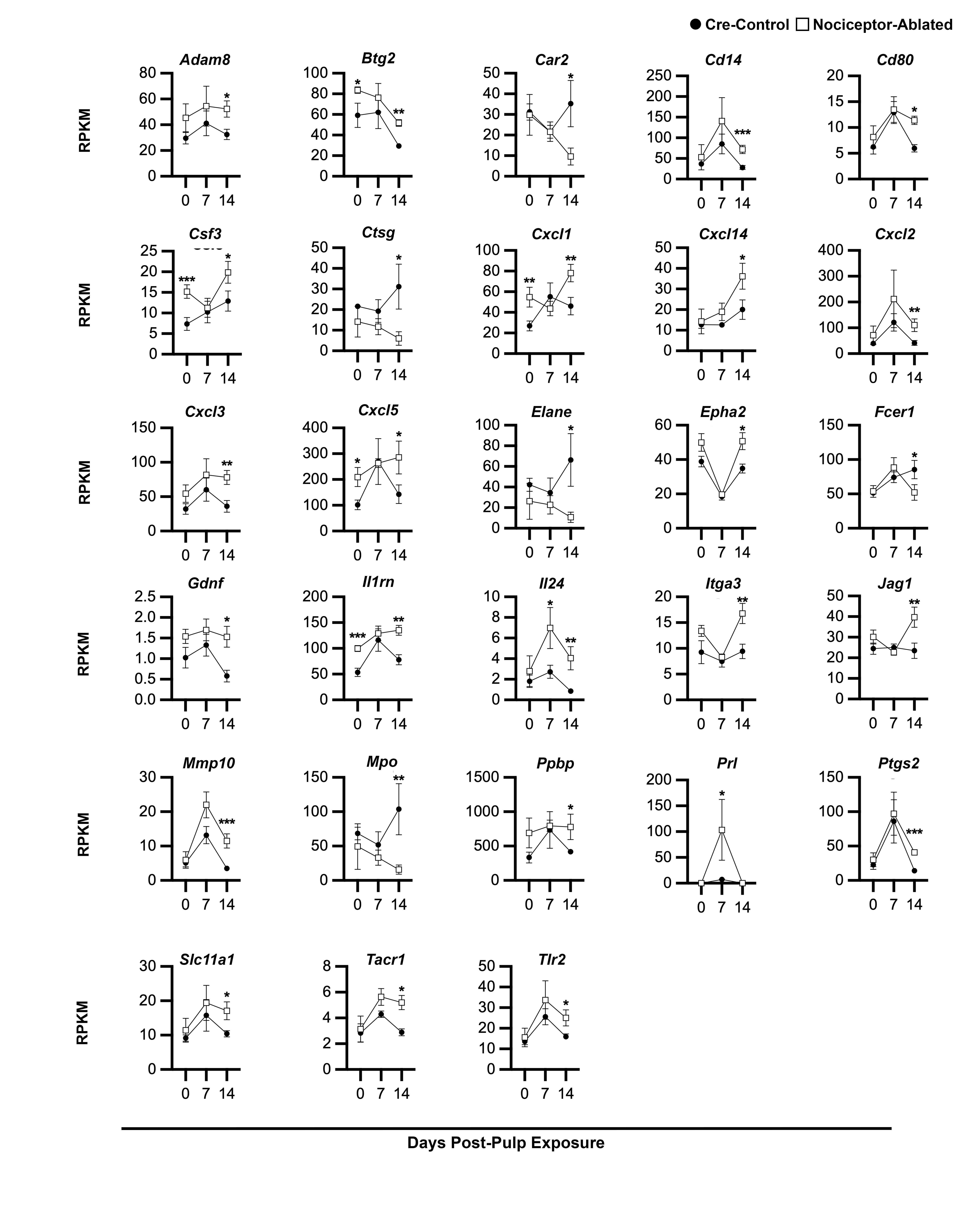
** **Supplementary Figure S2** Individual expression of selected Differentially Expressed Genes at 0, 7, and 14 days of infection are presented as Reads Per Kilobase of transcript per Million mapped (RPKM). The R package ‘DESeq’ was used to normalize data and find group-pairwise differential gene expression. *p<0.05, **p<0.001.

**
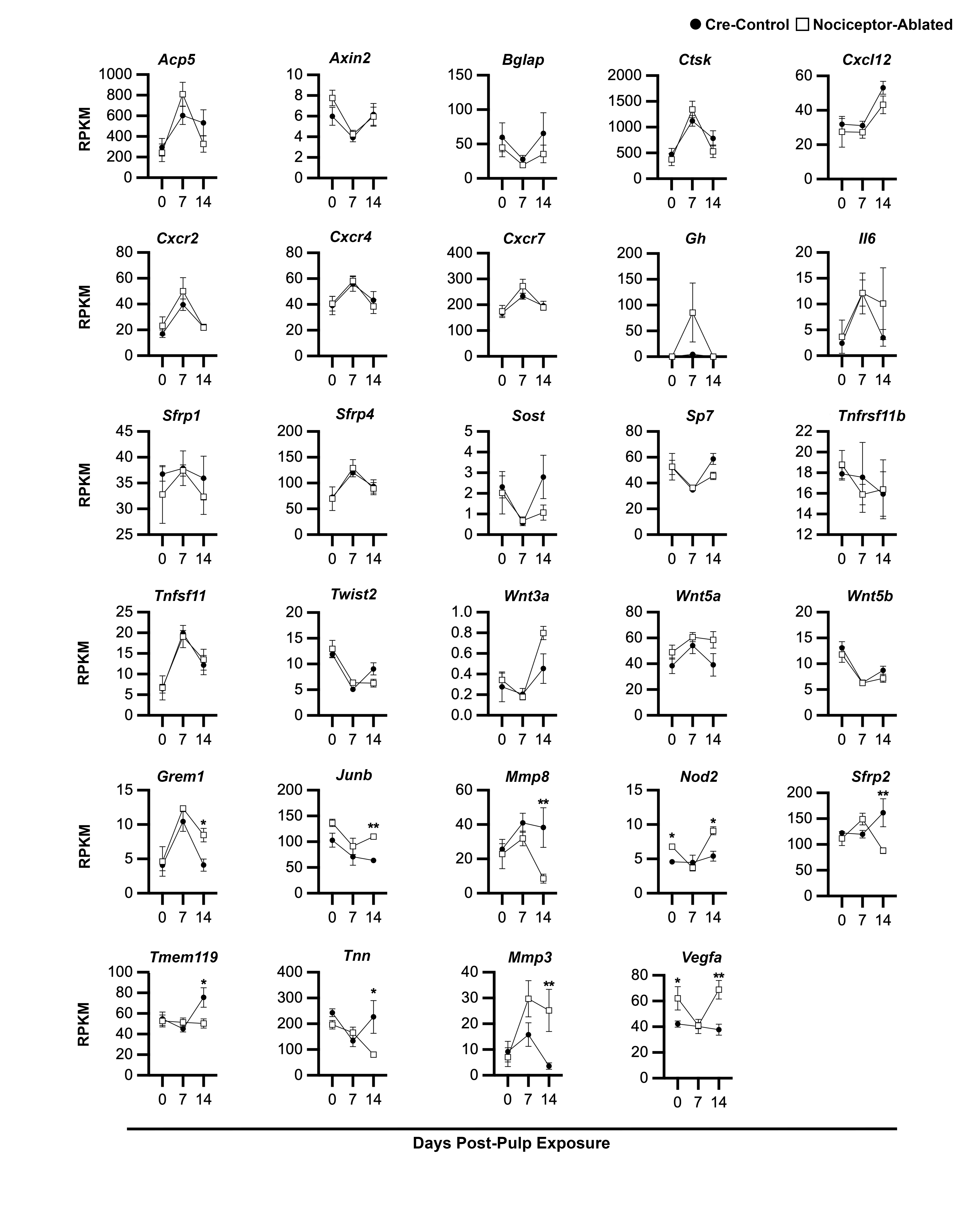
 Supplementary Figure S3** Individual expression of known inflammatory and bone metabolism genes at 0, 7, and 14 days of infection are presented as Reads Per Kilobase of transcript per Million mapped (RPKM). The R package ‘DESeq’ was used to normalize data and find group-pairwise differential gene expression. *p<0.05, **p<0.001.

**
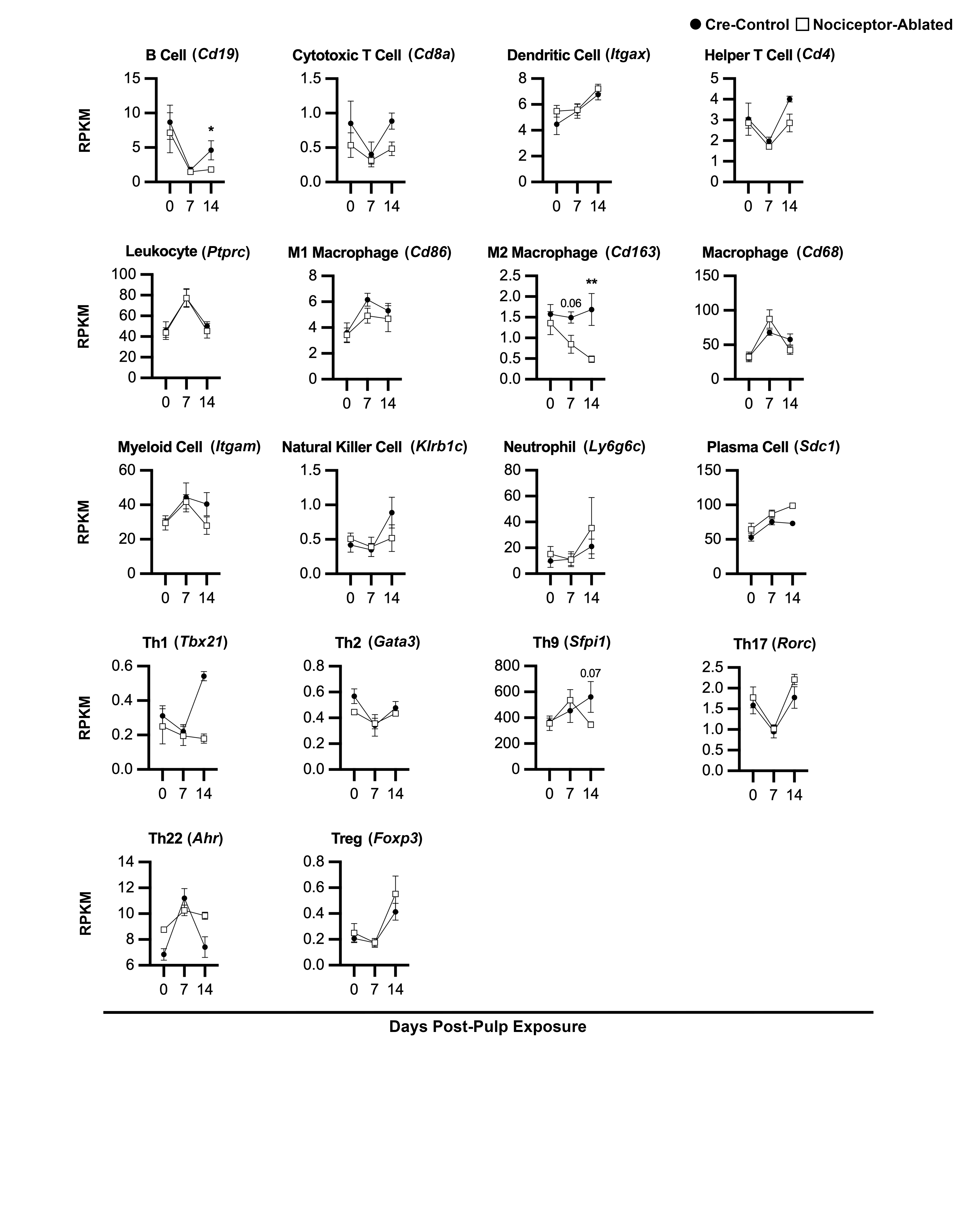
 Supplementary Figure S4** Individual expression of immune cell marker genes at 0, 7, and 14 days of infection are presented as Reads Per Kilobase of transcript per Million mapped (RPKM). The R package ‘DESeq’ was used to normalize data and find group-pairwise differential gene expression. *p<0.05, **p<0.001.
